# Reductions in malaria in pregnancy and adverse birth outcomes following indoor residual spraying of insecticide in Uganda

**DOI:** 10.1186/s12936-016-1489-x

**Published:** 2016-08-26

**Authors:** Mary K. Muhindo, Abel Kakuru, Paul Natureeba, Patricia Awori, Peter Olwoch, John Ategeka, Patience Nayebare, Tamara D. Clark, Atis Muehlenbachs, Michelle Roh, Betty Mpeka, Bryan Greenhouse, Diane V. Havlir, Moses R. Kamya, Grant Dorsey, Prasanna Jagannathan

**Affiliations:** 1 Infectious Diseases Research Collaboration, Kampala, Uganda; 2 Department of Medicine, University of California, San Francisco, USA; 3 Centers for Disease Control and Prevention, Atlanta, Georgia; 4 Uganda Indoor Residual Spraying Phase II Project, Abt Associates, Inc, Kampala, Uganda; 5 Department of Medicine, Makerere University College of Health Sciences, Kampala, Uganda

**Keywords:** Malaria in pregnancy, Placental malaria, *Plasmodium falciparum*, Indoor residual spraying, Vector-borne disease

## Abstract

**Background:**

Indoor residual spraying of insecticide (IRS) is a key intervention for reducing the burden of malaria in Africa. However, data on the impact of IRS on malaria in pregnancy and birth outcomes is limited.

**Methods:**

An observational study was conducted within a trial of intermittent preventive therapy during pregnancy in Tororo, Uganda. Women were enrolled at 12–20 weeks of gestation between June and October 2014, provided with insecticide-treated bed nets, and followed through delivery. From December 2014 to February 2015, carbamate-containing IRS was implemented in Tororo district for the first time. Exact spray dates were collected for each household. The exposure of interest was the proportion of time during a woman’s pregnancy under protection of IRS, with three categories of protection defined: no IRS protection, >0–20 % IRS protection, and 20–43 % IRS protection. Outcomes assessed included malaria incidence and parasite prevalence during pregnancy, placental malaria, low birth weight (LBW), pre-term delivery, and fetal/neonatal deaths.

**Results:**

Of 289 women followed, 134 had no IRS protection during pregnancy, 90 had >0–20 % IRS protection, and 65 had >20–43 % protection. During pregnancy, malaria incidence (0.49 vs 0.10 episodes ppy, P = 0.02) and parasite prevalence (20.0 vs 8.9 %, P < 0.001) were both significantly lower after IRS. At the time of delivery, the prevalence of placental parasitaemia was significantly higher in women with no IRS protection (16.8 %) compared to women with 0–20 % (1.1 %, P = 0.001) or >20–43 % IRS protection (1.6 %, P = 0.006). Compared to women with no IRS protection, those with >20–43 % IRS protection had a lower risk of LBW (20.9 vs 3.1 %, P = 0.002), pre-term birth (17.2 vs 1.5 %, P = 0.006), and fetal/neonatal deaths (7.5 vs 0 %, P = 0.03).

**Conclusion:**

In this setting, IRS was temporally associated with lower malaria parasite prevalence during pregnancy and at delivery, and improved birth outcomes. IRS may represent an important tool for combating malaria in pregnancy and for improving birth outcomes in malaria-endemic settings.

*Trial Registration* Current Controlled Trials Identifier NCT02163447

**Electronic supplementary material:**

The online version of this article (doi:10.1186/s12936-016-1489-x) contains supplementary material, which is available to authorized users.

## Background

In sub-Saharan Africa, over 30 million pregnancies occur annually in areas where malaria is endemic, and each year malaria in pregnancy is estimated to cause nearly one million low birth weight (LBW) deliveries and up to 100,000 infant deaths [1–3]. Given this high burden of disease, the World Health Organization (WHO) recommends the implementation of malaria preventive measures in all African countries where *Plasmodium falciparum* remains endemic, including the use of long-lasting, insecticide-treated nets (LLINs) and intermittent preventive treatment during pregnancy (IPTp) with sulfadoxine-pyrimethamine (SP) [4].

Despite these measures, rates of placental malaria and poor birth outcomes remain persistently high in many parts of Africa. In a recent cross-sectional study performed in Tororo, a highly endemic district in Uganda where nearly 70 % of pregnant women reported using a LLIN, the prevalence of placental malaria was 62 % in women who had received ≥two doses of IPTp-SP, and nearly 10 % of children were born with LBW [5]. This and other studies have raised concern for waning efficacy of LLINs given the emergence of resistance to pyrethroid insecticides [6–8], as well as waning efficacy of SP given widespread prevalence of drug-resistant parasites [9]. New interventions to prevent malaria during pregnancy and improve birth outcomes are urgently needed.

Indoor residual spraying (IRS) has been shown to be very effective in reducing vector densities, parasite prevalence and malaria morbidity in sub-Saharan Africa [10–14]. IRS can provide significant added protection together with LLINs compared to LLINs alone, particularly in areas with significant pyrethroid resistance [15–18], although evidence has been mixed [19, 20]. In 2006, Uganda began using IRS in selected districts, initially in the epidemic-prone areas of southwestern Uganda but later in the highly endemic areas of northern Uganda, with significant reductions in malaria morbidity and slide positivity rates [10, 14]. However, data quantifying the impact of IRS on malaria in pregnancy and birth outcomes are lacking.

Recently, a clinical trial was conducted comparing IPTp with dihydroartemisinin-piperaquine (DP) with IPTp-SP among HIV-uninfected pregnant women. This study was conducted in Tororo, Uganda, where, in separate studies, the East African International Centres of Excellence in Malaria Research (ICEMR) have been conducting cohort studies and entomology surveys since 2011 [21]. IPTp-DP was well tolerated, and significantly reduced parasite prevalence during pregnancy and the risk of placental malaria [22]. Midway through this trial, the President’s Malaria Initiative (PMI), through its implementing partner Abt Associates, initiated IRS in Tororo district for the first time [23]. Measures of malaria during pregnancy were compared before and after the implementation of IRS, and associations between IRS exposure during pregnancy and adverse birth outcomes were evaluated, among participants enrolled in this trial.

## Methods

### Study site and participants

Tororo is a rural district in southeastern Uganda with an entomologic inoculation rate estimated at 310 infectious bites per person year in 2012 [21]. Following a universal LLIN distribution campaign in November 2013 [23], 95 % of household in the region reported owning at least one LLIN [24]. Between June and October 2014, 300 women were enrolled into a double-blinded, placebo-controlled trial of three-dose SP vs three-dose DP vs monthly DP for IPTp. Details of the parent study have been described elsewhere [22]. Briefly, participants were HIV-uninfected pregnant women at least 16 years of age of all gravidities with an estimated gestational age of 12–20 weeks confirmed by ultrasound. The sub-study described in this report includes all women followed through delivery (n = 289).

From December 2014 to February 2015, Abt, in cooperation with the Ugandan Ministry of Health, initiated IRS in Tororo district using bendiocarb wettable powder, a carbamate insecticide [23]. Homes were sprayed once and following spraying, houses were marked with the date of spraying. Home visitors obtained the exact date of spraying from each participant’s household. If a household was not sprayed, the reasons for a lack of spraying were obtained and if the surrounding households in the village were sprayed, the date of spraying was obtained from surrounding homes.

### Study procedures and follow-up

At enrolment, women underwent a standardized examination and received a LLIN. Study participants were randomized in a 1:1:1 ratio to three-dose SP vs three-dose DP vs monthly DP for IPTp, as previously described [22]. Women received all of their medical care at a study clinic open daily. Routine visits were conducted every 4 weeks, including collection of dried blood spots (DBS). Women were encouraged to come to the clinic any time they were ill. Those who presented with a documented fever (tympanic temperature >38.0 °C) or history of fever in the previous 24 h had blood collected for a thick blood smear. If the smear was positive, the patient was diagnosed with malaria and treated with artemether-lumefantrine.

Women were encouraged to deliver at the hospital adjacent to the study clinic. Women delivering at home were visited by study staff at the time of delivery or as soon as possible afterwards. At delivery a standardized assessment was completed, including evaluation for birth weight and collection of specimens, including placental tissue and DBS of placental blood.

### Laboratory methods

DBS were tested for the presence of malaria parasites using loop-mediated isothermal amplification (LAMP) [25]. Placental tissues were processed for histological evidence of placental malaria as previously described [22]. Histopathology slides were read in duplicate using a standardized case record form by two independent readers and any discrepant results resolved by a third reader. Blood smears were collected in febrile women during pregnancy, stained with 2 % Giemsa and read by laboratory technologists. A blood smear was considered negative when the examination of 100 high power fields did not reveal asexual parasites. All slides were read by a second microscopist and a third reviewer settled any discrepant readings.

### Entomological surveys

Entomological surveys were conducted monthly beginning in June 2011 from 100 households enrolled in a separate longitudinal cohort study in Nagongera sub-county, Tororo district, as part of the ICEMR programme in Uganda [21]. Each month, miniature CDC light traps (Model 512; John W Hock Company, Gainesville, FL, USA) were positioned with the light 1 m above the floor at the foot end of the bed where a cohort study participant slept. Traps were set at 19.00 and collected at 07.00 h the following morning by field workers.

### Primary exposure variable

A woman was considered ‘protected under IRS’ if her house was directly sprayed, or if the surrounding village was sprayed, 14 days after spraying to account for the average incubation period for *P. falciparum*. The duration of pregnancy under protection of IRS was calculated as the date of delivery—(date of IRS + 14 days). The total duration of pregnancy was calculated as the date of delivery—estimated date of conception (ultrasound-confirmed). The proportion of time during a woman’s pregnancy that was under protection of IRS was then calculated as the duration of pregnancy under IRS/total duration of pregnancy. Three categories of protection were defined: no protection, >0–20 % of pregnancy protected, and >20–43 % of pregnancy protected.

### Outcomes

Outcomes assessed during pregnancy included parasite prevalence by LAMP and the incidence of malaria, calculated as the number of episodes per person years (ppy) at risk. Outcomes assessed at birth included the prevalence of parasitaemia at delivery by LAMP and evidence of placental malaria (parasites or pigment) by histopathology. Placental histopathology was also classified according to whether moderate-high grade pigment deposition was present (defined as pigment detected in >5 % of high power 40× fields) [26]. Birth outcomes assessed included birth weight, LBW (<2500 g), pre-term delivery (<37 weeks), and fetal/neonatal deaths, including spontaneous abortion, stillbirth and neonatal death within 4 weeks of delivery. For women giving birth to twins, delivery outcomes were based on whether the outcome was present in either child/placenta.

### Statistical methods

Data were double entered into an Access database. Data analysis was done using Stata version 14 (Stata Corp, College Station, TX, USA). Baseline characteristics between groups were compared using the $\chi^{2}$ test. Repeated prevalence measures and the daily risk of malaria during pregnancy were compared using generalized estimating equations with a log-binomial family, after adjustment for gravidity, age, IPTp arm, and gestational age when study drugs were started, and presented as adjusted risk ratios (aRR). For results stratified by IPTp arm, women randomized to either IPTp-DP arm were considered together. Dichotomous outcomes at delivery were compared using multivariate logistic regression. Continuous outcomes at delivery were compared using multivariate linear regression, with adjustment as above. As a secondary sensitivity analysis, propensity scores and inverse probability weighting were used to assess the average causal effect of IRS exposure with outcomes at delivery. All P-values were two-sided and values <0.05 considered statistically significant.

## Results

### Characteristics of study site and participants

**Fig. 1:**
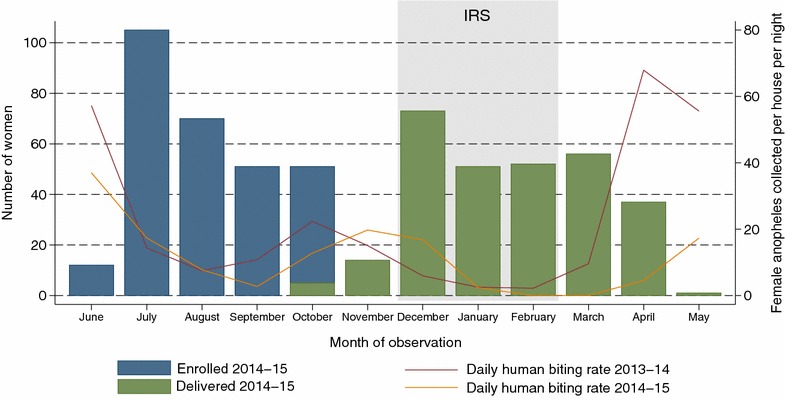
Temporal trends with enrolment, delivery and entomological measures. Shown are the number of women enrolled (*blue bars*) and number of women delivered (*green bars*) each month between 2014 and 2015. The *red line* (2013–2014) and *yellow line* (2014–2015) represent the number of female anopheline mosquitoes collected per household per night (*right y axis*) in Tororo district from 100 randomly selected households enrolled in the International Centers of Excellence in Malaria Research cohort [21]

Between June and October 2014, 300 women were enrolled prior to IRS, and 289 women were followed through delivery from October 2014 to May 2015 (Fig. 1). From December 2014 to February 2015, 123,924 of 145,574 households (85.1 %) in Tororo district received IRS. Among women in the study cohort, 95 % of households were sprayed between 1 December, 2014 and 31 January, 2015, with the remaining sprayed in February 2015. Monthly measurements of female *Anopheles* mosquitoes collected per household per night in Tororo were lower from February to May 2015, after IRS, compared with February–May 2014 (5.4 vs 33.7 female *Anopheles* mosquitoes per house per night, P < 0.001) (Fig. 1).

Of 289 women followed through delivery, 155 (53.6 %) were exposed to IRS before the birth of their child; 137 of these women’s households were directly sprayed, and 18 households (11.6 %) were indirectly protected through spraying of the surrounding village. Of the 134 women not exposed to IRS during pregnancy, six lived outside of the IRS spraying area in neighbouring Busia district, and 128 were exposed to IRS after the birth of their child. The median duration of pregnancy under protection of IRS was 46 days (IQR 20–68 days), and women were stratified into three groups based on the proportion of pregnancy under the protection of IRS: 0 (n = 134), >0–20 % (n = 90), and >20–43 % (n = 65). At enrolment, the prevalence of malaria parasites was >55 % and similar between the three groups (Table 1). Women with no IRS protection during pregnancy were slightly younger, more likely primigravid, and more likely to have study drugs initiated at 20 weeks gestation vs 16 weeks gestation, than women with IRS protection during pregnancy. IPTp treatment assignments, maternal weight gain, and household wealth were similar between groups (Table 1).

**Table 1:** Characteristics of women stratified by duration of pregnancy under the protection of IRS

Characteristic	Proportion of pregnancy under the protection of IRS		
	None (n = 134)	>0–20 % (n = 90)	>20–43 % (n = 65)
Malaria parasites detected at enrolment, n (%)	76 (56.7 %)	52 (57.8 %)	39 (60.0 %)
Age at enrolment in years, mean (SD)^a^	21.1 (3.9)	22.9 (4.0)	22.6 (4.0)
Gravidity, n (%)^a^			
1	64 (47.8 %)	23 (25.6 %)	16 (24.6 %)
2	37 (27.6 %)	25 (27.8 %)	25 (38.5 %)
>3	33 (24.6 %)	42 (46.7 %)	24 (36.9 %)
Study drugs started at 16 vs 20 weeks GA, n (%)^a^	61 (45.5 %)	85 (94.4 %)	57 (87.7 %)
Assigned IPTp arm, n (%)			
3-dose SP	45 (33.6 %)	34 (37.8 %)	23 (35.4 %)
3-dose DP	41 (30.6 %)	26 (28.9 %)	22 (33.9 %)
Monthly DP	48 (35.8 %)	30 (33.3 %)	20 (30.8 %)
Maternal weight gain during pregnancy, mean kg/week (SD)	0.25 (0.15)	0.25 (0.12)	0.24 (0.15)
Household wealth index, n (%)			
Lowest tertile	45 (33.6 %)	34 (37.8 %)	20 (30.8 %)
Middle tertile	45 (33.6 %)	23 (25.6 %)	27 (41.5 %)
Highest tertile	44 (32.8 %)	33 (36.7 %)	18 (27.7 %)

^a^P < 0.001

### Malaria incidence and parasite prevalence during pregnancy, before and after IRS

**Fig. 2:**
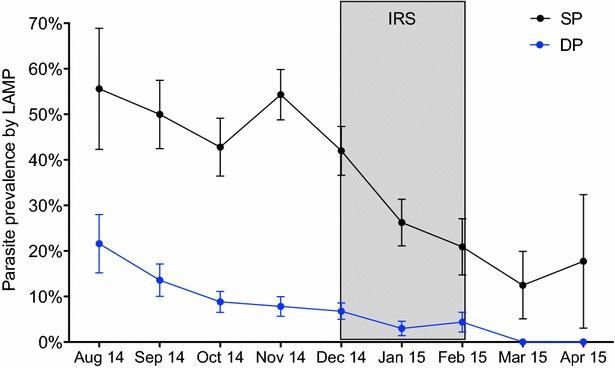
Parasite prevalence during pregnancy by calendar month before, during and after IRS. Shown is the predicted probability of having a positive (+) LAMP result during pregnancy, stratified by IPTp arm, after enrolment and initiation of study drugs, over the period of time of the study. Parasite prevalence point estimates and standard errors obtained using generalized estimating equations after adjustment for gravidity, age, and gestational age when study drugs were started. *Grey-shaded bar* shows the time period of IRS in Tororo district. SP (*black*): sulfadoxine-pyrimethamine; DP (*blue*): dihydroartemisinin-piperaquine

Parasite prevalence at enrolment and before study drug initiation was stable and high prior to IRS (see Additional file 1). After study drug initiation, the incidence of malaria during pregnancy was significantly higher before IRS (0.49 episodes ppy) compared to after IRS (0.10 episodes ppy, aRR 0.20, P = 0.02) after adjustment for gravidity, age, IPTp arm, and gestational age when study drugs were started. Similarly, the prevalence of malaria parasites by LAMP during pregnancy was significantly higher before IRS (20.0 %) compared to after IRS (8.9 %, aRR 0.40, P < 0.001). Declines were observed in women randomized to both IPTp-SP and IPTp-DP, although there was evidence of interaction between IPTp and IRS (P = 0.07, Fig. 2). Among women randomized to IPTp-SP, the prevalence of parasitaemia was 44.0 % before IRS, before declining to 22.0 % after IRS (aRR 0.49, P = 0.003). Among women randomized to IPTp-DP, parasite prevalence was 8.7 % before IRS vs 1.1 % after IRS (aRR 0.13, P = 0.003). Women living in households directly sprayed vs those indirectly sprayed were both protected by IRS. Among women living in households directly sprayed, parasite prevalence was 20.2 % before IRS vs 8.5 % after IRS (aRR 0.42, 95 % CI 0.26–0.70, P = 0.001). Among women living in households indirectly sprayed, parasite prevalence was 34.2 % before IRS vs 5.0 % after IRS (aRR 0.12, 95 % CI 0.03–0.59, P = 0.009), consistent with community-wide benefits of IRS on parasitaemia [11].

### Association between IRS and placental malaria

At delivery, the prevalence of malaria parasites in placental blood by LAMP was significantly higher in women with no IRS protection (16.8 %) compared to women with >0–20 % (1.1 %, P = 0.001) or >20–43 % IRS protection during pregnancy (1.6 %, P = 0.006) (Table 2). The prevalence of any placental malaria by histopathology, as well as the prevalence of moderate to high-grade pigment deposition, was higher in women with no IRS protection compared with women with IRS protection (Table 2), but the differences were not statistically significant. All 105 placentas that were positive for placental malaria by histopathology had pigment in fibrin that was indicative of past infection, and seven (11 %) had parasites indicative of concomitant active infection. All seven with active infection were not exposed to IRS prior to delivery.

**Table 2:** Associations between proportion of pregnancy under the protection of IRS and outcomes measured at birth

Outcome	Proportion of pregnancy under the protection of IRS		
	None	>0–20 %	>20–43 %
Placental blood positive for malaria parasites by LAMP^a^			
Risk	22/131 (16.8 %)	1/88 (1.1 %)	1/61 (1.6 %)
aOR^b^ (95 % CI)	Reference group	0.03 (0–0.25)	0.05 (0.01–0.41)
*P* value		0.001	0.006
Any evidence of placental malaria by histopathology^a^			
Risk	63/132 (47.7 %)	25/88 (28.4 %)	17/62 (27.4 %)
aOR^b^ (95 % CI)	Reference group	0.77 (0.35–1.69)	0.63 (0.27–1.48)
P value		0.51	0.29
Moderate-high grade pigment deposition by histopathology^a^			
Risk	37/132 (28.0 %)	11/88 (12.5 %)	9/62 (14.5 %)
aOR^b^ (95 % CI)	Reference group	0.52 (0.20–1.36)	0.54 (0.20–1.47)
P value		0.18	0.23
Risk	28/134 (20.9 %)	9/90 (10.0 %)	2/65 (3.1 %)
LBW (<2500 g)			
aOR^b^ (95 % CI)	Reference group	0.29 (0.12–0.75)	0.08 (0.02–0.39)
P value		0.01	0.002
Risk	23/134 (17.2 %)	3/90 (3.3 %)	1/65 (1.5 %)
Pre-term delivery (<37 weeks)			
aOR^b^ (95 % CI)	Reference group	0.13 (0.03–0.53)	0.05 (0.01–0.43)
P value		0.005	0.006
Fetal/neonatal deaths			
Risk	10/134 (7.5 %)	1/90 (1.1 %)	0/65 (0 %)
aOR^b^ (95 % CI)	Reference group	0.10 (0–0.86)	0 (n/a)
P value		0.03	0.03

*LBW* low birth weight; *aOR* adjusted odds ratio

^a^Includes all subjects with evaluable outcomes of interest

^b^Odds ratio adjusted for gravidity, household wealth, presence of parasites at enrollment, gestational age study drugs started, and assigned IPTp treatment arm

### Association between IRS and birth outcomes

**Fig. 3:**
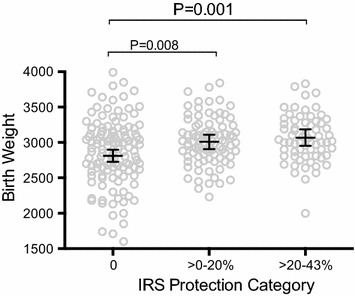
Impact of IRS on birth weight. Shown are birth weights (*clear circles*) from infants born to mothers with no IRS exposure (n = 134), >0–20 % exposure (n = 90), and >20–43 % exposure (n = 65). Also shown are model adjusted means and 95 % CI (*lines* and *error bars*), and P values comparing birth weights between groups, adjusted for gravidity, gestation age study drugs started, wealth category, LAMP at enrolment, and treatment arm

The prevalence of LBW was significantly higher in infants born to mothers with no IRS protection (20.9 %) compared to those with >0–20 % (10.0 %, P = 0.01) or >20–43 % IRS protection (3.1 %, P = 0.002) (Table 2). Compared to infants born to women with no IRS protection, mean birth weight was 196 g higher among infants born to women with 0–20 % IRS protection (95 % CI 51–340 g, P = 0.008) and 257 g higher among infants born to women with >20–43 % IRS protection (95 % CI 105–409 g, P = 0.001) (Fig. 3). When restricting the analysis to women without histologic evidence of placental malaria, the prevalence of LBW was similarly higher in infants born to mothers with no IRS protection (18.8 %) compared to those with >0–20 % (7.9 %, P = 0.07) or >20–43 % IRS protection (2.2 %, P = 0.03).

The prevalence of pre-term birth was significantly higher in infants born to women with no IRS protection (17.2 %) compared to those with >0–20 % (3.3 %, P = 0.01) or >20–43 % IRS protection (1.5 %, P = 0.006) (Table 2). Of 11 fetal/neonatal deaths (three spontaneous abortions, three still births, and five neonatal deaths), ten (7.5 % of pregnancies) occurred among those with no IRS protection, and none occurred among those with 20–43 % IRS protection (P = 0.03). Given the imbalances noted between IRS protection strata, a sensitivity analysis was performed to evaluate associations between IRS and outcomes at delivery using propensity scores with inverse probability weighting, with similar results (see Additional file 2). In all analyses, there was no significant interaction observed between IRS and assigned IPTp group.

## Discussion

While conducting a clinical trial comparing novel IPTp strategies in a high-transmission setting in Uganda with near-universal LLIN coverage, the unexpected opportunity arose to evaluate the additional impact of IRS on measures of malaria during pregnancy and adverse birth outcomes. In this setting, the period following protection with IRS was associated with a significantly lower incidence of symptomatic malaria and prevalence of parasitaemia during pregnancy. Reductions in parasite prevalence were observed among women randomized to either IPTp-SP or IPTp-DP, suggesting additional benefits of IRS when given along with LLINs and IPTp. Importantly, the period following protection with IRS was also associated with a significantly lower prevalence of malaria parasites in placental blood at the time of delivery, and a significantly lower risk of LBW, pre-term delivery, and fetal/neonatal deaths.

WHO recommends IRS as a central part of malaria control policy in Africa, but coverage rates have been low (<10 %) and have declined in recent years, possibly due to increased costs from spraying with non-pyrethroid insecticides [4]. Although IRS has been shown to be very effective in reducing vector densities and malaria morbidity, particularly among children [10–18], no study has assessed its impact on malaria morbidity during pregnancy and birth outcomes. In eastern Uganda, rates of pyrethroid resistance among *Anopheles* mosquitoes and the prevalence of molecular markers of SP resistance among *P. falciparum* parasites are very high [6, 27]. This may explain the high parasite prevalence prior to IRS among pregnant women given LLINs and randomized to IPTp-SP, as well as the significant reduction in parasite prevalence both during pregnancy and at delivery following implementation of carbamate-containing IRS. Although significant differences in placental histopathology with increasing IRS exposure were not observed, it is possible that histopathologic changes may reflect early malaria exposure during pregnancy [28], as all women became pregnant prior to the implementation of IRS, and nearly 60 % of women had evidence of asymptomatic parasitaemia at enrolment [22].

Notably, IRS was associated with significant reductions in the risk of LBW and pre-term delivery, and no fetal or neonatal deaths occurred among women with >20 % IRS protection during pregnancy. LBW and pre-term delivery are multifactorial disorders, and relatively few interventions have been shown to improve these outcomes, especially in resource-limited settings [29–31]. It is possible that IRS may have led to improved birth outcomes by preventing placental infection by malaria parasites [2, 32]. However, malaria in pregnancy is thought to contribute to 20 % of LBW in sub-Saharan Africa [2], significantly less than the reductions observed in this study, and improvements in birth outcomes were observed even in women without histological evidence of placental malaria. Furthermore, not all interventions that have been shown to prevent malaria infection during pregnancy have been shown to improve birth outcomes [33]. In addition to targeting *Anopheles* mosquitoes and preventing malaria, IRS may be targeting other vectors such as, potentially, *Aedes* and culicine mosquitoes, argasid ticks and fleas that are capable of transmitting a variety of vector-borne pathogens [34] that could be associated with poor pregnancy outcomes. However, data on additional pathogens that may have been prevented by IRS were unavailable in this study.

There are several limitations to this study. As this was a pre-post observational study nested within a trial of IPTp, definitive causal inferences regarding the effect of IRS on pregnancy and birth outcomes cannot be drawn. Unmeasured confounders, including temporal and seasonal changes, may have affected these results, although parasite prevalence prior to IRS was stable and high, lessening this possibility. There were also significant differences between the IRS exposure groups, including a higher proportion of first pregnancies among women in the group not exposed to IRS during pregnancy. Multivariate adjustment and a secondary sensitivity analysis using propensity scores and inverse probability weighting were performed to adjust for these differences, although it is possible that residual confounding remains. Furthermore, it is possible that given the timing of IRS within the context of this trial, a cohort effect may have led to these results, specifically with regard to birth outcomes. However, women were enrolled over a period of 6 months, and delivered over a period of 6 months, reducing the possibility that this form of bias was responsible for these results. Finally, due to sample size and study design, several aspects which would further help to inform policy were incompletely addressed. These include a determination of effect duration, as well as the relative contributions to protection from direct household *versus* community spraying.

## Conclusions

The provision of IRS, in combination with LLINs and IPTp, was temporally associated with a reduction in the incidence of malaria during pregnancy, prevalence of malaria parasites during pregnancy and in placental blood, as well as improved birth outcomes. IRS may represent an important tool for combating malaria in pregnancy and for improving birth outcomes in malaria-endemic settings. Additional and larger controlled evaluations, including monitoring for malaria in pregnancy metrics in settings where IRS is currently being implemented, are warranted to further assess this promising strategy, and to inform policy.

## Additional files

10.1186/s12936-016-1489-x Parasite prevalence during pregnancy by calendar month at enrolment and visits prior to study drug initiation. Shown is the predicted probability of having a positive (+) LAMP result during pregnancy at enrolment and before initiation of study drugs. Parasite prevalence point estimates and standard errors obtained using generalized estimating equations after adjustment for gravidity and age.
10.1186/s12936-016-1489-x Sensitivity analysis: estimation of effect of any IRS *vs* no IRS protection on outcomes measured at birth.
