# Supplementary material for: Reductions in malaria in pregnancy and adverse birth outcomes following indoor residual spraying of insecticide in Uganda
Source: Malar J. 2016 Aug 26;15(1):437. doi: 10.1186/s12936-016-1489-x (PMC5002129; doi:10.1186/s12936-016-1489-x)
Supplement: Supplementary file 1 — 10.1186/s12936-016-1489-x Parasite prevalence during pregnancy by calendar month at enrolment and visits prior to study drug initiation. Shown is the predicted probability of having a positive (+) LAMP result during pregnancy at enrolment and before initiation of study drugs. Parasite prevalence point estimates and standard errors obtained using generalized estimating equations after adjustment for gravidity and age. [file 12936_2016_1489_MOESM1_ESM.docx]

**Supplementary Table. Sensitivity Analysis: Estimation of effect of any IRS vs no IRS protection on outcomes measured at birth**

| **Outcome** | **Marginal effect of IRS from multivariate logistic model^b^** | | | **Causal effect of IRS using logistic model with inverse-probability weights^c^** | | |
| --- | --- | --- | --- | --- | --- | --- |
|  | **Average estimated marginal effect** | **95% CI** | **p-value** | **Average estimated causal effect** | **95% CI** | **p-value** |
| Placental blood positive for malaria parasites by LAMP^a^ | -17.3 | -24.5 – -10.1 | <0.001 | -17.0 | -24.5 – -9.5 | <0.001 |
| Any evidence of placental malaria by histopathology^a^ | -5.5 | -16.6 – 5.6 | 0.333 | -8.6 | -19.7 – 2.5 | 0.128 |
| LBW (< 2500 gm) | -17.0 | -26.3 – -7.6 | <0.001 | -15.7 | -26.4 – -5.1 | 0.004 |
| Preterm delivery (< 37 weeks) | -15.0 | -22.8 – -7.2 | <0.001 | -17.8 | -26.2 – -9.4 | <0.001 |
| Fetal/Neonatal Deaths | -8.7 | -14.7 - -2.6 | 0.005 | -6.7 | -11.1 – -2.4 | 0.003 |

LBW: Low birth weight; aOR: adjusted odds ratio; IPW: inverse probability weighting; CI: Confidence interval

^a^ Includes all subjects with evaluable outcomes of interest

^b^ Average marginal effect: Estimated absolute risk difference between any IRS vs. no IRS, adjusted for gravidity, household wealth, presence of parasites at enrollment, gestational age study drugs started, and assigned IPTp treatment arm

^c^ Average causal effect: Estimated absolute risk difference between Any IRS vs. no IRS using inverse probability weighting
