# Supplementary figures and images for: Reductions in malaria in pregnancy and adverse birth outcomes following indoor residual spraying of insecticide in Uganda
Source: Malar J. 2016 Aug 26;15(1):437. doi: 10.1186/s12936-016-1489-x (PMC5002129; doi:10.1186/s12936-016-1489-x)

Parasite prevalence by LAMP

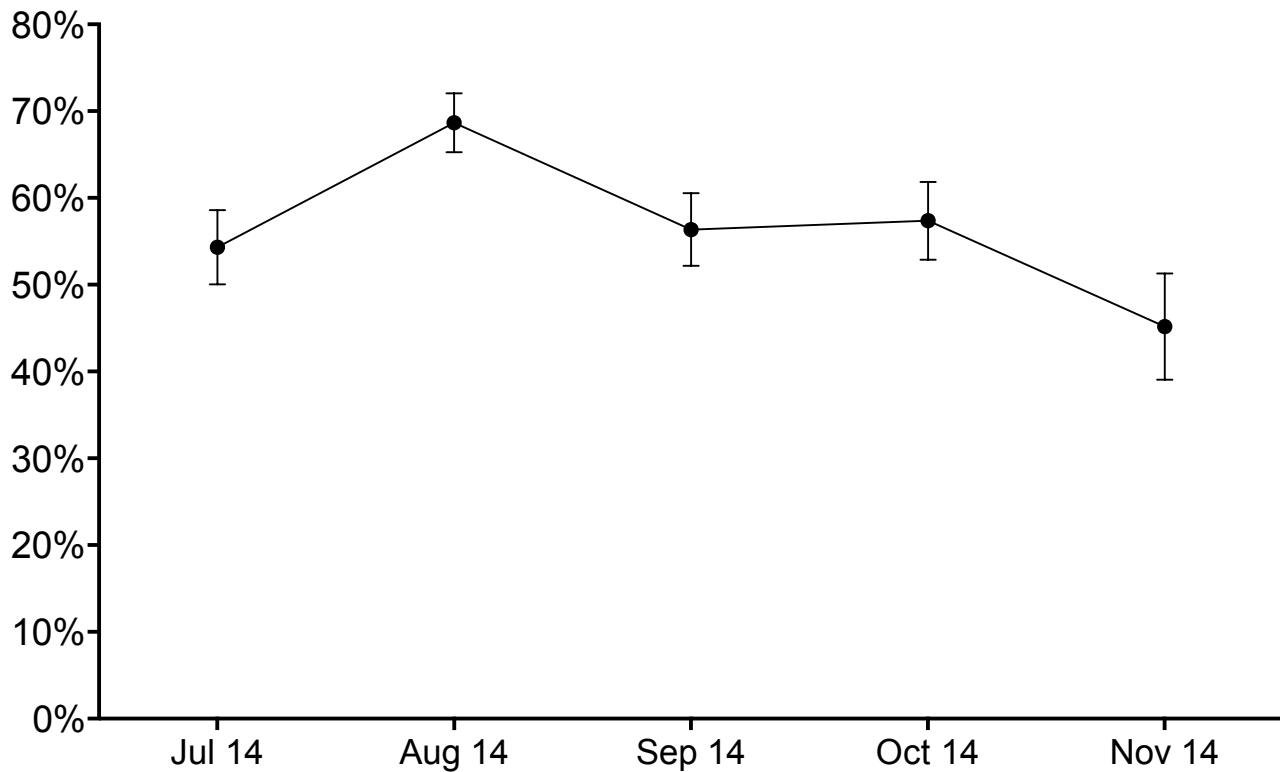

Supplement: Supplementary file 2 — 10.1186/s12936-016-1489-x Sensitivity analysis: estimation of effect of any IRS vs no IRS protection on outcomes measured at birth. [file 12936_2016_1489_MOESM2_ESM.pdf]
